# New Insight on Human Type 1 Diabetes Biology: nPOD and nPOD-Transplantation

**DOI:** 10.1007/s11892-014-0530-0

**Published:** 2014-08-21

**Authors:** Alberto Pugliese, Francesco Vendrame, Helena Reijonen, Mark A. Atkinson, Martha Campbell-Thompson, George W. Burke

**Affiliations:** 1 Diabetes Research Institute, Miller School of Medicine, University of Miami, 1450 NW 10th Avenue, Miami, FL 33136 USA; 2 Division of Diabetes, Endocrinology and Metabolism, Department of Medicine, Miller School of Medicine, University of Miami, Miami, FL USA; 3 Department of Immunology and Microbiology, Miller School of Medicine, University of Miami, Miami, FL USA; 4 Department of Surgery, Miller School of Medicine, University of Miami, Miami, FL USA; 5 Benaroya Research Institute, Seattle, WA USA; 6 Department of Pathology, Immunology, and Laboratory Medicine, College of Medicine, University of Florida, Gainesville, FL USA; 7 Department of Pediatrics, College of Medicine, University of Florida, Gainesville, FL USA

**Keywords:** Type 1 diabetes, Transplantation, Pancreas, nPOD

## Abstract

The Juvenile Diabetes Research Foundation (JDRF) Network for Pancreatic Organ Donors with Diabetes (JDRF nPOD) was established to obtain human pancreata and other tissues from organ donors with type 1 diabetes (T1D) in support of research focused on disease pathogenesis. Since 2007, nPOD has recovered tissues from over 100 T1D donors and distributed specimens to approximately 130 projects led by investigators worldwide. More recently, nPOD established a programmatic expansion that further links the transplantation world to nPOD, nPOD-Transplantation; this effort is pioneering novel approaches to extend the study of islet autoimmunity to the transplanted pancreas and to consent patients for postmortem organ donation directed towards diabetes research. Finally, nPOD actively fosters and coordinates collaborative research among nPOD investigators, with the formation of working groups and the application of team science approaches. Exciting findings are emerging from the collective work of nPOD investigators, which covers multiple aspects of islet autoimmunity and beta cell biology.

## Introduction

Type 1 diabetes (T1D) is considered a T cell-mediated autoimmune disease leading to the chronic destruction of pancreatic beta cells. The disease is often diagnosed in childhood or adolescence, but is also common in adult individuals [1]. Despite significant advances in the identification of autoantigens and susceptibility genes, most of the clinical trials conducted so far have resulted in no to modest impact on preservation of insulin secretion after diagnosis [2]. These outcomes may reflect to a significant extent our incomplete knowledge of etiological factors and pathogenic mechanisms [1, 2], which is also a consequence of the rare access to the pancreas and other disease-related tissues from patients. Furthermore, existing data from human pancreata are largely from older studies that could not take advantage of the sophisticated modern technologies available today. Thus, the field has relied largely on experimental rodent models of the disease, especially the nonobese diabetic (NOD) mouse [3], to investigate the disease pathogenesis. This approach has advantages, but also several limitations, given the rodent models only partially reproduce the human condition. Moreover, several key questions cannot be easily investigated in experimental animals, such as epitope specificity, key phenotypic and functional features of autoreactive T and B cells, the possible presence of viral infections, potential pathways of beta cell regeneration, and importantly, yet uncovered pathogenic mechanisms that may play a significant role in disease pathogenesis beyond T cell-mediated islet cell destruction. Progress in these areas could lead to the identification of novel therapeutic targets and guide strategies for modulating multiple disease pathways, both immune and non-immune, in future clinical trials. To promote research about the pathogenesis of human T1D and address the above needs, beginning in 2007, the Juvenile Diabetes Research Foundation (JDRF) supported the creation of the JDRF Network for the Pancreatic Organ Donors with Diabetes (JDRF nPOD; www.JDRFnPOD.org).

The JDRF nPOD has three main strategic goals:

1. Recover organs from deceased donors with T1D (diagnosed or sub-clinical) and establish a repository of pancreas and other relevant tissues (pancreatic lymph nodes, spleen, thymus, blood, and others)
2. Distribute donor specimens to approved investigators, worldwide, to support comprehensive and diversified studies of human T1D
3. Promote and coordinate collaboration and team science approaches, by establishing and managing project interactions and focused working groups, to achieve the most comprehensive understanding of human T1D

This article provides an overview of the JDRF nPOD, both in terms of its operational models and the discoveries of nPOD investigators that have been recently contributed to the scientific literature.

### The JDRF nPOD Operational Model

**Fig. 1:**
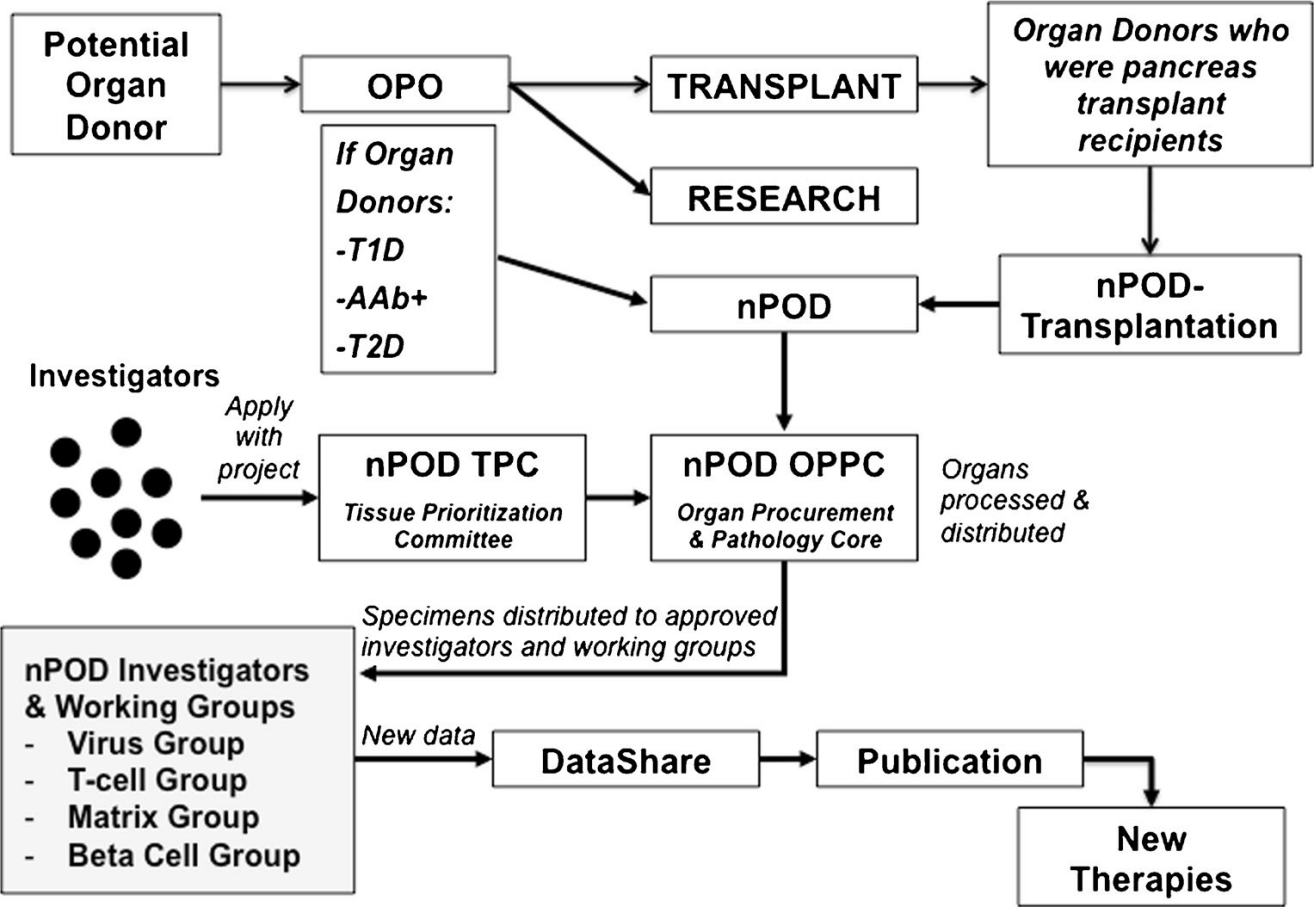
Operational scheme of the JDRF nPOD. The scheme illustrates interaction with the organ procurement organization to identify organ donors relevant to nPOD research and the nPOD-Transplantation model that allows pancreas transplant recipients to consent for organ donation to nPOD to support T1D research. Tissues are distributed to investigators and working groups to support advances in research and hopefully the discovery of novel therapeutic targets

The nPOD operational model and standard operating procedures (SOP) have been recently described in detail [4, 5] and are illustrated in Fig. 1. In the USA, nPOD staff members work with over 50 organ procurement organizations (OPOs), tissue banks, and medical examiners, directly or indirectly, through referral organizations such as the International Institute for the Advancement of Medicine and National Disease Research Interchange, to obtain referrals of organ donors. Organs offered to nPOD are accepted if processing can begin within 24 h of cold ischemia time (since the organ is cooled with a cold preservation solution) to ensure the highest quality of the tissues for cutting-edge research applications. The nPOD Organ Processing and Pathology Core (OPPC) in Gainesville, Florida, processes tissues according to SOP (available at http://www.jdrfnpod.org/for-investigators/standard-operating-procedures/) [5–7]. Tissues and their samples are inventoried and baseline characterization performed including DNA and RNA quality and pancreas histopathology before distribution to investigators worldwide.

nPOD actively seeks organ donors with T1D, with particular emphasis on those with shorter disease duration that are more likely to still have detectable islet autoimmunity and residual insulin-positive beta cells. Recent onset T1D may sometimes be diagnosed when a patient has developed diabetic ketoacidosis (DKA) and has been admitted to an emergency room; nPOD established a partnership with the Pediatric and Critical Care sections of the American College of Emergency Physicians (ACEP), to engage pediatric physicians in the referral of thankfully rare but unfortunate deaths resulting from complications of DKA [8, 9].

To identify donors who might have had asymptomatic prediabetes, characterized by islet immune infiltrates (insulitis) in the absence of diabetes symptoms, nPOD has enabled several OPOs to screen non-diabetic donors for autoantibodies associated with T1D risk. nPOD provides the OPO clinical laboratories with training, protocols, and reagents so that their staff can rapidly (3 h) screen donors for autoantibodies to the GAD65, IA-2, and ZnT8 autoantigens (a customized assay kit developed by Dr. Clive Wasserfall from standardized ELISAs (KRONUS, Boise, ID)).

The nPOD repository currently includes specimens from 100 donors with T1D, including donors with recent onset diagnosis, and from 22 non-diabetic donors with islet autoantibodies. At least 19 donors have been identified with insulitis in their pancreas, including donors of pediatric age. An updated list of donors is maintained on the nPOD website (http://www.jdrfnpod.org/for-investigators/donor-groups/). nPOD also recovers donors with type 2 diabetes (T2D), as a comparison to T1D donors and to study pathways of beta cell physiology and pathology that can also be relevant to T1D. Finally, nPOD recovers organs from donors affected with gestational diabetes and cystic fibrosis; non-diabetic donors are also accepted as controls.

### nPOD Investigators and Collaborative Working Groups

Investigators interested in accessing nPOD tissues submit a description of the scientific project they intend to carry out using donor specimens. The project is reviewed by either an academic or industry Tissue Prioritization Committee (TPC), depending on the investigator’s affiliation. The nPOD TPC will typically approve projects that have scientific merit and are feasible, and often will provide critical guidance and constructive feedback, both in terms of scientific design and strategic considerations that take into account sample availability and relevance to the study. Investigators are also prompted to embrace nPOD policies about data sharing and collaboration (described below). Indeed, once a project is approved, investigators are encouraged to take an active role in nPOD research activities, beyond the conduct of their own study. In fact, they are made aware of collaborative opportunities within the network, either with specific investigators or with working groups. The nPOD staff promotes interactions among investigators by organizing periodic web-based meetings (webinars) to review data and discuss scientific projects or provide training on the use of the online pathology system. In addition, nPOD holds an annual investigators’ meeting where data, most often unpublished, are presented. Indeed, nPOD is implementing “real-time” data sharing to best take advantage of the coordinated analyses of donor specimens by multiple investigators. Thus, investigators are given the opportunity to share results prior to publication, among members of the nPOD network, and this facilitates changes in the experimental strategy, reconciliation of discordant findings, as well as case selection during the course of a project. Data sharing is made possible by the creation of *DataShare*, a collaborative web-based tool for data upload and communication [4].

Moreover, nPOD has developed collaborative working groups focused on major research areas where questions are many and complex and will likely require a team science approach for significant progress to be made. Thus, interested investigators begin webinar discussions of a major topic and collectively define critical questions to address in the context of a collaborative experimental strategy where synergies can be exploited. For example, nPOD has established working groups on viral etiology (nPOD-Virus), autoreactive T cells (nPOD-T), and extracellular matrix components (nPOD-Matrix), and a group on beta cells (nPOD-Beta Cell) is developing. Since inception, nPOD has provided tissue specimens in support of about 130 projects led by investigators around the world. nPOD-supported projects have a broad scope and cover critical research areas relevant to T1D, including islet autoimmunity, environmental factors, beta cell physiology and dysfunction, as well as pancreas development and remodeling, with emphasis on beta cell regeneration, trans-differentiation, and dedifferentiation. nPOD samples are analyzed with diverse and advanced methodological approaches for immunohistochemistry, immunofluorescence, electron microscopy, proteomics genotyping, RNA sequencing, gene expression, multi-parameter flow cytometry, and more.

### The nPOD-Transplantation Initiative

Studies we conducted at the University of Miami in T1D recipients of simultaneous pancreas-kidney (SPK) transplants have revealed that approximately 5–6 % of such patients will, on long-term follow-up, develop recurrence of T1D in the pancreas allograft [10, 11]. While this concept was demonstrated in the mid-1980 in identical twins or HLA identical recipients of segmental pancreas grafts from living-related donors that were not or were only minimally immunosuppressed [12–15], our studies show that disease recurrence may occur despite immunosuppression that prevents rejection and largely resembles the features of T1D [11]. Indeed, we have identified several patients in whom we have shown the following:

1. On post-transplant follow-up, reappearance of autoantibodies, months to more often years prior to diabetes recurrence, correlating with increased risk of diabetes recurrence [11, 16, 17]
2. At presentation of diabetes recurrence, severe, insulin-requiring hyperglycemia, in the absence of rejection and stable exocrine pancreas (urine amylase) and kidney (serum creatinine) graft function, yet in the presence of selective loss of C-peptide secretion
3. In the pancreas transplant biopsy, the presence of insulitis and/or beta cell loss, at the same time when islet cell autoantibodies and autoreactive T cells are present in the circulation and in the pancreas transplant-associated lymph nodes [11]
4. The presence of autoreactive T cells in the circulation that correlated with disease activity and progression; in fact, in patients that received additional immunosuppression in the attempt to salvage the residual B cell mass demonstrated at biopsy, autoreactive T cells were no longer detected after treatment but reappeared on later follow-up. Their return was followed by further and complete loss of C-peptide [11]. Moreover, we found that autoreactive T cells in the circulation were often present in the pancreas transplant lymph node (unpublished).
5. The demonstration that antigen-specific autoreactive T cells isolated from these patients could mediate beta cell destruction when transplanted with human islets in immunodeficient mice. Moreover, the T cell receptor sequences (V-beta and CDR3) in samples collected over time suggested a memory phenotype [11, 18] and more recent phenotypic studies show that these autoreactive T cells are memory cells, including in the insulitis lesion (unpublished).

**Fig. 2:**
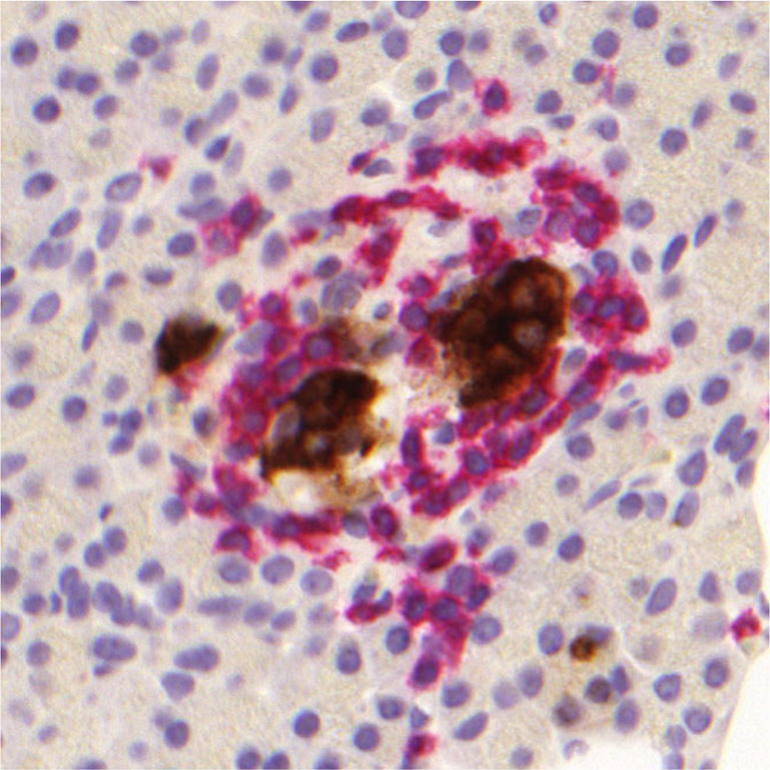
Example on insulitis in the transplanted pancreas from a patient who had developed recurrent islet autoimmunity despite immunosuppression. Pancreatic beta cells are stained for insulin (*brown*); infiltrating lymphocytes are stained for CD3 (*red*)

Importantly, the biopsies of the transplanted pancreas in our cases with diabetes recurrence revealed insulitis and beta cell loss that did not appear dissimilar in severity and pathological features from nPOD donors with spontaneous disease in whom insulitis is present in their native pancreas. Figure 2 shows an example of insulitis in the transplanted pancreas from a patient with recurrence of T1D. Thus, biopsies from transplanted patients could help us better understand human disease and could help pinpoint molecular targets expressed by autoreactive T and B cells. An important consideration is that patients who develop T1D recurrence appear to carry memory autoreactive lymphocytes that are reactivated after transplantation and many years after the onset of the original disease. It would seem likely that these cells might represent highly disease relevant populations, and thus, studying their antigen specificity and functional/phenotypic features could help us identify important therapeutic targets. Moreover, detailed clinical history and therapeutic regimens are fully documented and can support full interpretation of research data. Therefore, we believe that tissues from immunosuppressed, transplanted patients can potentially be of critical importance in addressing key questions about T1D and for developing new means to cure it. Given the relative difficulty of identifying organ donors with active islet autoimmunity and insulitis, specimens from these patients represent an important additional source of research samples for the nPOD community.

Studies of biopsies from pancreas transplant recipients with recurrent disease can also be relevant to study beta cell regeneration and other mechanisms of pancreas remodeling. Pancreas transplant biopsies from several of our patients with recurrent autoimmunity revealed the presence of ductal cells expressing insulin [19]. These cells expressed the pancreatic-duodenal homeobox-1 transcription factor (PDX-1). In one patient, the one with the most severe B cell loss in islets (virtually complete), we detected rare insulin+CK-19+PDX-1+ cells expressing Ki-67, indicating proliferation. In the same patient, some insulin+CK-19+ ductal cells expressed chromogranin A, suggesting further endocrine differentiation. While the patient remained insulin-dependent after the development of recurrent diabetes, these findings provide evidence for a mechanism of tissue remodeling and B cell regeneration involving ductal cells in the human transplanted pancreas. Other studies have shown similar changes in human pancreata with chronic autoimmune pancreatitis [20], chronic pancreatitis [21], in streptozotocin-treated nonhuman primates [22] and in various rodent models [23]. It is possible that chronic immunosuppression, as given to transplant recipients, could allow for some level of beta cell replication in the native pancreas, but addressing this question has proven difficult in recent clinical studies [24] and may require studies of the native pancreas if this can be recovered from immunosuppressed transplant recipients.

In 2012, based on the above considerations, nPOD initiated a programmatic expansion, called nPOD-Transplantation, in which patients with T1D who are recipients of pancreas transplants consent for directed, postmortem donation to the nPOD program. This new program is pioneering an approach that allows T1D recipients of pancreas allografts to consent for postmortem donation of both their transplanted and native pancreata to nPOD, so that their organs can support diabetes research (Fig. 1). We currently have a quite high acceptance rate, with approximately 85 % of our transplant recipients consenting to postmortem donation. Recovery would then be possible through OPOs and tissue banks. We believe that eventually the opportunity for postmortem donation should be offered to all patients with T1D who desire to direct organ donation in support of diabetes research. With growing demand for tissue from investigators, such an approach would enhance nPOD’s ability to recover more relevant cases with more clinical detailed medical history. With several large clinical research studies enrolling thousands of patients and relatives, interfacing with such initiatives would further the nPOD mission and ultimately advance diabetes research.

Moreover, the nPOD-Transplantation program is implementing means to recover both the native and transplanted pancreas not just after passing, but through performing biopsy of living patients as well in relation to clinical course and relevance to patient care. Accordingly, nPOD-Transplantation has since inception obtained and made available to investigators pancreas transplant biopsies from several patients with diabetes recurrence, and showed that insulitis was also present. There are currently five transplant cases deposited in the nPOD repository with collaborative and coordinated investigations presently ongoing, which cover several key questions related to disease pathogenesis.

### New Findings from nPOD-Supported Studies

During the last few years, many papers have reported novel findings based on the study of nPOD samples [25–27, 28•, 29, 30, 31••, 32–38, 39•, 40, 41, 42•, 43, 44•, 45, 46•, 47•]. Major findings emerging from these studies are summarized below:

1. A key question posed when nPOD was established was whether individuals with autoantibody (single or multiple) would have insulitis in the pancreas [26, 48, 49]. To date, insulitis has been demonstrated in two of three non-diabetic donors with multiple autoantibodies but not in any of the 18 donors with a single autoantibody. Although such donors likely did not have a relative with T1D and rarely were found to carry high-risk HLA types for T1D, these findings are in agreement with the analysis of tissue blocks from pancreata that were processed for islet isolation in Europe [49] and with the well-established low risk of T1D demonstrated by prospective follow-up of patients’ relatives with a single autoantibody [50]. However, a number of studies are discovering evidence for abnormalities affecting the pancreas of donors with autoantibodies, perhaps pointing at some initial stages in the disease pathogenesis that may or may not necessarily progress to overt disease but could be important co-factors in their own right or if more sustained autoimmunity was triggered.
2. Examination of nPOD specimens also shows that human insulitis is rarely as severe as reported in the NOD mouse, an experimental model that presents striking similarity with the human disease [3], even in newly diagnosed patients. This is consistent with the cases previously reported in the literature [51] and in biopsies recently obtained at onset from living, newly diagnosed patients from the DiVid study in Norway [52]. A group of nPOD investigators has recently come together and conducted an extensive pathology review of T1D cases from historical cases and contemporary nPOD cases, and have reported a consensus definition of human insulitis that is hoped will provide consistency for interpretation and data comparison across future studies [53••]. Another important finding is that insulitis may be present in some cases for many years after diagnosis [31••], further supporting the concept that islet autoimmunity is a chronic process even after diagnosis, which was raised in earlier studies [54]. This is also consistent with the finding that a significant proportion of patients express autoantibodies for many years after diagnosis, as shown for example in transplant recipients when tested before transplantation [55]. In our cohort, approximately 40 % of patients expressed one or more autoantibodies at the time of transplantation [17].
3. Studies on nPOD cases with insulitis reveal that islet autoantigen-specific CD8 T cells are present in the insulitis lesion, using MHC tetramers to identify such cells in the pancreas of nPOD donors. These findings place those autoreactive T cells at the crime scene and as such provide as much causal evidence as is possible through the study of pathology specimens. Thus, those autoreactive T cells and antigen specificities can be considered validated therapeutic targets [31••]. Furthermore, the study produced initial evidence for an evolution of the inflammatory lesion. In fact, islets from pancreata with recent diagnosis were infiltrated by autoreactive CD8 T cells with single antigen specificity, while pancreata from patients with longer disease duration and insulitis had autoreactive CD8 T cells with multiple antigen specificities. Such a dynamic evolution may reflect epitope spreading of the autoimmune response as disease advances, possibly sustained by progressive release of novel antigens that follow beta cell death.
4. Another critical area of study deals with how the immune system loses tolerance to islet cell autoantigens. The discovery that insulin and other self-molecules are expressed in the thymus in early life linked this mechanism to tolerance, and genetically determined influences of levels of autoantigen expression were linked to T1D risk and enhanced probability of generating autoreactive T cells [56–59]. In the thymus, expression of self-molecules has been ascribed to thymic epithelial cells and bone marrow-derived antigen-presenting cells [56, 60, 61] and largely depend on the *Aire* transcription factor, also known as the autoimmune regulator [62]. Following the studies in thymus, cells capable of expressing self-molecules and mediate immune tolerance were described in peripheral lymphoid tissues as well [60]. The analysis of pancreatic lymph nodes showed that self-molecule genes are expressed at reduced levels in T1D compared to non-diabetic nPOD donors [25]; such a reduction appears dependent on the induction of alternative splicing of the Deaf1 transcription factor, which is also linked to the transcription of self-molecule genes. Alternative splicing of Deaf1 may be caused by inflammation, which in turn impairs expression of the eukaryotic translation initiation factor 4 gamma 3 (Eif4g3) [43]. These studies illustrate molecular events that may result in lower expression of self-molecules (e.g., insulin, an autoantigen in T1D) [63] and impaired induction of peripheral tolerance at a site that is believed to be key to the regulation and activation of islet autoimmune responses, the pancreatic lymph node. nPOD also supported studies that provided a more in-depth phenotypic analysis of cells that express self-molecules in peripheral lymphoid tissues, in particular extra-thymic *Aire*-expressing cells [64] that are now known to represent a bone marrow-derived, distinct phenotype with similarities to dendritic cells [46•].
5. nPOD studies show that beta cell loss occurs with distinct patterns, indicating that the disease pathogenesis is likely heterogeneous and that more than one pathway may explain beta cell death [27]. However, beta cell loss is not absolute and, in several patients insulin-positive beta cells and glucose transporters [29], are detectable for many years after diagnosis [31••]. These results, together with the persistence of insulitis, further support the concept that the disease process is chronic; importantly, these findings challenge the traditional view that beta cell loss is virtually complete at the time of clinical onset, as also suggested by a meta-analysis of previously published cases [65, 66]. Importantly, evidence for significant dysfunction of pancreatic beta cells is emerging from unpublished data from the new onset patients in the DiVid study [52] and from our own pancreas transplant recipients with disease recurrence, in which impaired beta cell function is shown despite the presence of insulin-positive cells at biopsy. These findings concur with recent reports showing that C-peptide secretion can persist in many patients, albeit at low levels, for several years after diagnosis [28•, 67•, 68•]. Altogether, these findings suggest the hypothesis that some patients might perhaps benefit from therapy to alter disease course or promote beta cell regeneration beyond just a few months after diagnosis; there is now a trend towards conducting clinical trials in patients with longer disease duration which will inform us as to whether there is indeed a wider window of therapeutic opportunity.
6. Novel molecules have now been associated with disease, including selected chemokines [38]. Key mediators of endoplasmic reticulum stress were found in islets from T1D donors, such as C/EBP homologous protein (CHOP); the immunoglobulin heavy chain (BIP) was found expressed in insulin-positive islets with insulitis [36]. Changes in key constituents of the extracellular matrix were also noted, such as hyaluronan, a glycosaminoglycan that was dramatically upregulated with the islet and outside endocrine cells juxtaposed to microvessels in nPOD donors with T1D; hyaluronan was also present around infiltrating cells in cases with insulitis. Moreover, hyaluronan-binding proteins such as inter-α-inhibitor, versican, and tumor necrosis factor-stimulated gene-6 showed accumulation in hyaluronan-rich areas in pancreatic islets with insulitis. Hyaluronan and IαI amassed in follicular germinal centers and in T cell areas in lymph nodes and spleens in T1D compared to controls. These patterns were only observed in tissues from younger donors with disease duration of less than 10 years. Furthermore, hyaluronan and inter-α-inhibitor were also detected at increased levels in lymph nodes and spleens (follicular germinal centers and T cell areas) of T1D donors compared to controls [47•]. Importantly, these extracellular matrix components have been linked to lymphocyte adhesion and migration and to islet inflammation [32, 69]. Cathepsins were identified as key mediators in the degradation and loss of integrity of the peri-islet basement membrane, which allow autoreactive T cells to penetrate and infiltrate pancreatic islets [42•]. The cytokine IL-15 and its receptor (IL-15Rα) were found perturbed in the pancreatic islets and serum of patients; of note, treatment with tofacitinib to manipulate this pathway resulted in diabetes reversal in NOD mice, providing rationale from both nPOD specimens and experimental models for testing this as a new therapeutic option in clinical trials [44•]. A recent study reported that C4d is deposited in T1D pancreata [45], specifically in the blood vessel endothelium and in the extracellular matrix around exocrine ducts and blood vessels.
7. The nPOD-Virus group is conducting extensive studies seeking to address key questions about the role of viruses in T1D etiology. Ongoing analysis of the nPOD samples is providing additional evidence for an association of enteroviruses with T1D [39•]. Importantly, signs of enteroviral infection are seen also in those nPOD donors with a longer duration of disease; this finding may be explained by persistence of a viral infection or by the occurrence of multiple infections over time. Considering the other findings pointing at chronicity, any of these two infection patterns may contribute to chronicity and help explain the patchy distribution of insulitis and beta cell loss. Ultimately, more robust information about which viruses are more prevalent and a better characterization of the type of infection may provide support for vaccine development and clinical trials.
8. nPOD investigations are exploring the extent to which beta cell replication might be possible in the human pancreas. Beta cell replication appears more sustained in early life. While replication is possible in adult patients, it is rare [35]. However, in nPOD donors with T2D, treatment with incretins was associated with the presence of insulin-glucagon double hormone-positive cells; these might represent a transitional cell type reflecting regeneration or pancreas remodeling [41].
9. The analysis of the pancreas body weight revealed that T1D pancreata have reduced weight compared to those of non-diabetic donors. Importantly, the pancreas weight was also reduced in donors with autoantibodies [34]. These findings suggest the hypothesis that exocrine abnormalities might exist in the T1D pancreas, resulting in reduced weight, and that such abnormalities may even precede T1D development. If so, the reduction of the pancreas weight may not simply be a consequence of pancreatic atrophy, related to insulin deficiency, as until recently believed.

## Conclusions

In closing, the JDRF nPOD is actively promoting the study of T1D focusing research on the analysis of pancreas and other relevant tissues from patients. Through its interactions with OPO and transplant programs, nPOD and nPOD-Transplantation provide investigators with the rare opportunity to study specimens with ongoing disease and compare findings at different time points in the natural history of the disease and after transplantation, when the immunosuppression cover may provide additional insight into the immune responses and possibly regeneration. nPOD is pioneering an expanded team science approach, involving the coordination of collaborative efforts and real-time data sharing in the joint study of a cohort of T1D donors. Studies supported by nPOD are shedding new light into the complexity and heterogeneity of the human disease and is challenging the concept that beta cell destruction is completed and autoimmunity almost exhausted within a few months from diagnosis. nPOD can also help in validating therapeutic targets and in this function contribute to defining strategic direction and priority for clinical trials to interdict T1D.
